# Sex differences in G protein-coupled estrogen receptor-mediated mechanisms in preclinical models of anxiety and fear

**DOI:** 10.3389/fnbeh.2025.1655725

**Published:** 2025-11-11

**Authors:** AnBinh S. Tran, Lisa Y. Maeng

**Affiliations:** 1 Department of Psychology, University of Massachusetts Boston, Boston, MA, United States; 2 Department of Psychology, Developmental and Brain Sciences Program, University of Massachusetts Boston, Boston, MA, United States

**Keywords:** GPER, estradiol, GPR30, fear, anxiety, stress, sex differences

## Abstract

Sex differences are well-documented in the prevalence of psychiatric disorders, with anxiety and stress-related conditions more common in women. Growing evidence highlights the role of sex hormones, particularly estradiol (E2), and its receptor mechanisms as contributing factors to this disparity. Estrogen exerts its effects through three main receptors: estrogen receptor alpha (ERα), estrogen receptor beta (ERβ), and the G protein-coupled estrogen receptor (GPER). While the classical receptors ERα and ERβ have been widely studied in the context of fear and anxiety, the role of GPER remains less understood. Moreover, estrogen receptors themselves may be sexually dimorphic, adding complexity to their functional roles. Preclinical research has been valuable in advancing our understanding of these mechanisms; therefore, this review mostly focuses on findings from rodent studies. Here we discuss the influence of sex and E2 on anxiety and fear-related behavior, highlight emerging research on sex differences in GPER modulation of fear and anxiety in mice, rats, and humans, and explore GPER as a potential therapeutic target for anxiety and stress-related disorders.

## Introduction

Estrogens, particularly 17β-estradiol (E2), are steroid hormones that significantly influence the neurobiological mechanisms underlying emotional learning and memory. E2, the most potent form of estrogen, is present in both sexes, but most research on its effects on these processes has been conducted in females. Emerging evidence suggests that E2 may exert sex-specific effects, potentially contributing to observed sex differences in the prevalence of anxiety and stress-related disorders, such as post-traumatic stress disorder (PTSD). For instance, low E2 levels have been associated with heightened anxiety in women, whereas elevated E2 levels in men have been linked to increased depressive symptoms (Stanikova et al., 2018).

E2 acts through classical genomic pathways via nuclear estrogen receptors such as estrogen receptor alpha (ERα) and estrogen receptor beta (ERβ), as well as rapid, non-genomic mechanisms involving membrane-bound receptors. One such receptor, the G protein-coupled estrogen receptor (GPER), notable for its distinct structure and role in neurophysiology, has been implicated in the modulation of anxiety, fear behaviors, stress responses, and memory consolidation– crucial processes in the pathophysiology of PTSD and other anxiety-related disorders. This review examines current preclinical findings on E2’s role in mediating sex differences in stress- and fear-related behaviors, focusing on GPER’s potential involvement and its sex-specific mechanisms.

### Sex and estradiol influences on animal behavior

Various well-established behavioral paradigms have been used to model fear and anxiety in rodents, providing insights into the mechanisms underlying conditions such as PTSD and anxiety disorders. Sex differences have been reported in the behavioral outcomes of these paradigms and appear to be sensitive to E2’s modulatory effects. These paradigms highlight how sex hormones, particularly E2, influence fear and anxiety responses.

#### Elevated plus maze test

The elevated plus maze (EPM) test measures anxiety-like behavior by assessing a rodent’s exploration of open arms versus enclosed arms, with more time spent in the open arms suggesting lower anxiety (Pellow et al., 1985). Adult female Wistar rats spend more time and make more open arm entries than males, indicating less anxiety-like behavior (Johnston and File, 1991; Knight et al., 2021). Circulating gonadal hormones may mediate these sex differences. Female rats in proestrus spent significantly more time in the open arms of the EPM than male rats, OVX rats, or rats in other estrous stages. E2 administration to rats in diestrus also increased open-arm time (Marcondes et al., 2001), suggesting an anxiolytic effect.

#### Open field test and light–dark test

The open field test (OFT) and light–dark test (LDT) are also used to assess anxiety behavior. In the LDT, spending more time in the dark compartment suggests higher anxiety (Bourin and Hascoët, 2003), whereas in the OFT, more time spent in the center versus the periphery indicates reduced anxiety (Ohl, 2003). Sex differences and gonadal hormone influences have been noted in anxiety-related behaviors in the OFT (Blizard et al., 1975). Knight et al. (2021) reported that adult female Wistar rats traveled more to and spent more time in the center of the OF than males. In addition, female mice given E2 subcutaneously spent significantly more time in the OFT center and significantly more time in the LDT lit area (Walf and Frye, 2010), indicating anxiolytic E2 effects.

#### Fear conditioning and extinction

Fear conditioning is a paradigm for investigating mechanisms underlying fear control, which is impaired in PTSD and anxiety (Milad and Quirk, 2012; Zoladz et al., 2012). Learning how to reduce or regulate fear responses once a threat is removed, a process known as fear extinction, has been integral to treatments for fear-related disorders, while poor extinction learning and memory are characteristic of PTSD (Milad and Quirk, 2012).

E2 levels influence sex differences in fear extinction. High E2 female rats display greater fear extinction retention in a manner similar to males, and both outperform low E2 females (Milad et al., 2009). In contextual and auditory fear conditioning, male rodents have exhibited stronger conditioned fear acquisition compared to female rodents (Maren et al., 1994), which was unaffected by castration (Anagnostaras et al., 1998). Alternatively, ovariectomized (OVX) female rats have shown enhanced fear expression, suggesting E2 may reduce fear expression (Gupta et al., 2001). Additionally, females in proestrus and estrus have displayed more rapid rates of successful fear extinction compared to males and diestrus females. E2-treated OVX females had more rapid rates of successful fear extinction when compared to control and OVX females treated with progesterone (Chang et al., 2009). Together, these data highlight E2’s capability to regulate fear.

#### Single prolonged stress model

A preclinical model used to study neurobiological mechanisms underlying PTSD is the single prolonged stress (SPS) paradigm. Rodents are subjected to a sequence of stressors, including forced swim, restraint stress, and anesthesia, followed by a post-stress incubation period. After SPS, rodents show enhanced fear responses and impaired fear extinction, similar to symptoms observed in PTSD patients (Liberzon et al., 1997). Female rats administered an estrogen receptor antagonist before SPS did not exhibit the typical SPS-induced impairment in extinction (Biddle and Knox, 2023). E2-treated SPS females also showed no change in freezing levels during extinction training, whereas E2 reduced freezing in non-SPS control rats (Biddle and Knox, 2023). These data indicate that E2 can modulate SPS-induced effects on fear extinction (Keller et al., 2015).

Together, these findings support a modulatory role of E2 in fear and anxiety. E2 contributes to sex differences observed in fear extinction and anxiety-like behavior and may confer resilience against stress-induced impairments, particularly in females. This highlights the importance of investigating the receptor mechanisms underlying E2’s effects in both sexes.

### GPER’S discovery, mechanism, signaling effects, and function

The classical genomic nuclear receptors, ERα and ERβ, have been extensively studied for their role in modulating anxiety and fear behaviors (Cover et al., 2014; Borrow and Handa, 2017). However, the G protein-coupled receptor, also known as GPER and GPR30 (Alexander et al., 2008), is the focus of this review due to its recent implication in these processes. GPER was first identified and cloned in the 1990s (O'Dowd et al., 1998; Carmeci et al., 1997) and was found to mediate E2’s rapid non-genomic effects (Filardo et al., 2000; Filardo et al., 2002). The selective GPR30 agonist, G1, and selective antagonists, G15 and G36, were developed between 2006 and 2011 (Bologa et al., 2006; Dennis et al., 2009; Dennis et al., 2011), and they have been integral to understanding GPER function.

Diverging from classical ERα and ERβ genomic mechanisms, GPER activates pathways via a nongenomic mechanism (Iqbal et al., 2024) and initiates rapid transcriptional responses, including quick activation of ion channels and second messenger pathways within seconds to minutes. Once an agonist, such as G1, binds to the receptor, G proteins divide into subunits Gα and Gβγ (Luo et al., 2023) as well as Gαi/o and Gq/11 proteins (Bushi et al., 2025). Gα activates adenylyl cyclase (AC), an enzyme that converts adenosine triphosphate into cyclic adenosine monophosphate (Thomas et al., 2005), which then activates protein kinase A (PKA), an enzyme regulating cellular processes (Thomas et al., 2005). In contrast, Gαi/o suppresses AC activity, resulting in lower cAMP levels and reduced activation of PKA, while simultaneously stimulating the phosphatidylinositol 3-kinase/protein kinase B signaling pathway, aiding cell survival, growth, and metabolism (Bushi et al., 2025). Gβγ recruits a steroid receptor coactivator, which activates metalloproteinases and causes the release of heparin-binding epidermal growth factors (HB-EGFs) from the cell surface (Prossnitz et al., 2008). These HB-EGFs transactivate an EGF receptor, resulting in PI3K/Akt, ERK1/2, and MAPK pathway activations (Filardo et al., 2000; Bustamante-Barrientos et al., 2021; Bushi et al., 2025). Gβγ also participates in regulation of potassium and calcium ion flow across the cell membrane, processes critical for cellular excitability, function, and responsiveness. Additionally, PLCβ is activated by Gβγ, enhancing inositol trisphosphate (IP3) and diacylglycerol (DAG) production. The Gq/11 signaling pathway activates phospholipase C, which also catalyzes the formation of IP3 and DAG. IP3 facilitates the release of calcium ions from the endoplasmic reticulum into the cytosol, increasing intracellular calcium and initiating calcium-dependent activities. Concurrently, DAG activates protein kinase C, which phosphorylates target proteins involved in functions such as secretion, gene transcription, and cell proliferation (Bushi et al., 2025).

Sex differences in GPER signaling pathways have been described. GPER activation enhances object recognition (OR) and spatial memory performance in gonadectomized (GDE) male and female mice via different signaling mechanisms. Behavioral effects of GPER involved C-Jun N-terminal kinase (JNK) signaling in the dorsal hippocampus (DH) in females; however, CREB levels, but not JNK, were increased in the DH in males, indicating sex-specific signaling pathways in the DH (Kim et al., 2016; Machado et al., 2024).

GPER activity can also impact physiological and hormonal stress responses. Inhibition of GPER via G15 treatment has been shown to prevent the rapid, non-genomic effects of corticosterone (CORT) in the infralimbic region of the medial prefrontal cortex in male mice (Karst and Joëls, 2023). Given the sexually dimorphic nature of the hypothalamic–pituitary–adrenal (HPA) axis (Heck and Handa, 2019), GPER may also play a role in mediating sex differences in stress-induced CORT responses. For example, GPER-knockout (GPER-KO) female rats exhibited significantly lower basal serum CORT levels compared to wild-type females, and this difference varied across the estrous cycle, suggesting estrous phase-dependent GPER effects on basal CORT (Zheng et al., 2020). Zheng et al. (2020) also found that after acute restraint stress exposure, GPER-KO females showed an increased adrenocorticotropic hormone (ACTH) response compared to WT females, an effect that was more pronounced in females than males, indicating a sex-dependent role for GPER in stress reactivity.

### GPER localization within the brain

**Figure 1:**
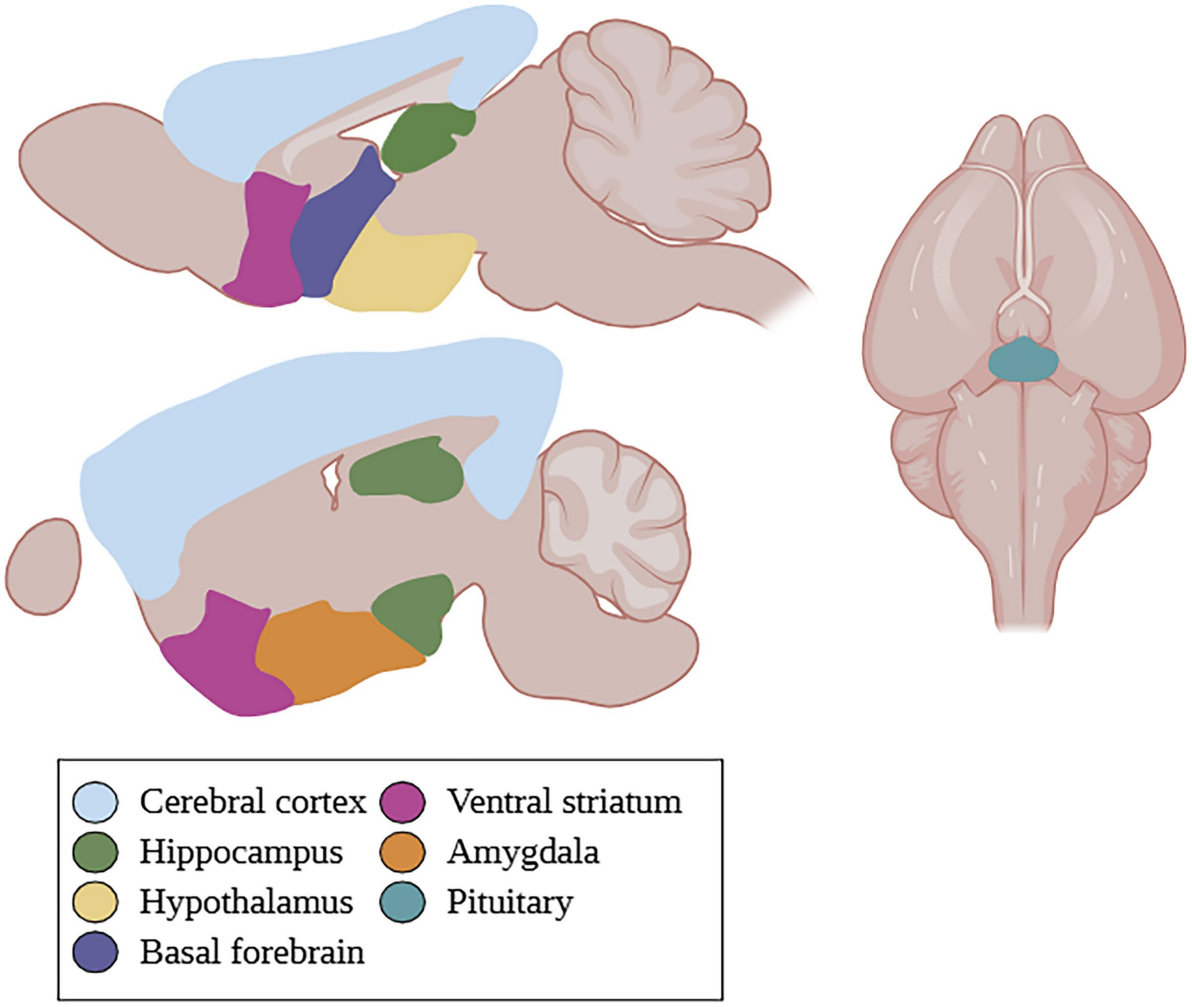
GPER localization in the rodent brain. GPER is found in the cerebral cortex, hippocampus, hypothalamus, basal forebrain, ventral striatum, amygdala, and pituitary. Created in BioRender.com.

GPER is widely expressed in various tissues and cell types. It is found in the hippocampus, cerebral cortex, hypothalamus, striatum, and amygdala of the central nervous system (Luo et al., 2023). More specifically, it can be found in the perirhinal cortex, pituitary, substantia nigra, basolateral amygdala, as well as the cholinergic neurons of the basal forebrain (Hammond and Gibbs, 2011; Hazell et al., 2009; Tian et al., 2013; Figure 1). It is also widely distributed on the subcellular level, with reports of expression in the cell membrane, endoplasmic reticulum, Golgi complex, and nucleus (Luo et al., 2023).

GPER distribution also appears to be sex-specific (Llorente et al., 2020; Hutson et al., 2019). GPER immunoreactive cells were higher in adult male Wistar rats than females in the posterodorsal medial amygdala and specific subdivisions of the CA1-CA3 and dentate gyrus of the DH (Llorente et al., 2020); however, GPER expression was higher in the basolateral amygdala of females compared to males. Importantly, GPER expression appeared to differ in subregions of both the amygdala and DH across the estrous phases in females (Llorente et al., 2020). These findings suggest that estradiol modulation via GPER activation in the limbic areas, amygdala, and DH may be sex- and estrous cycle-dependent.

## Animal studies—mice

### GPER’S role in fear behavior

GPER’s role in fear memory appears to be sex- and age-dependent. In middle-aged male mice, GPER expression is significantly reduced in the hippocampus, the structure playing a key role in learning and memory (Xu et al., 2018). Subcutaneous GPER agonist G1 treatment for 15 days in intact male and female middle-aged mice improved contextual and cued fear memory in a dose-dependent manner through the activation of brain-derived neurotrophic factor/tropomyosin receptor kinase B (BDNF/TrkB) signaling, but not in 2-month-old male mice (Xu et al., 2018). Although GPER activation appears to generally enhance memory consolidation in both sexes, age may limit its effects on contextual and cued fear memory processes in male mice.

GPER knockout revealed sex differences in fear behavior, with GPER-KO female mice freezing more than males when returned to the conditioning context after contextual fear conditioning (Koitmäe et al., 2023). Freezing was also higher in GPER-KO female mice in the high E2 estrous cycle phase compared to those in low E2 phases. Further, there was enhanced long-term potentiation in GPER-KO female mice and increased spinophilin expression in the hippocampus of low E2 GPER-KO female mice (Koitmäe et al., 2023). These findings suggest that GPER activity enhances contextual fear memory consolidation in both sexes, but its absence reveals sex differences and estrous phase-dependent changes in hippocampal synaptic plasticity and fear behavior.

### GPER’S role in anxiety behavior

Findings on GPER’s role in anxiety-related behaviors in mice are mixed, with some studies suggesting that GPER activation increases anxiety, whereas others demonstrate anxiety-reducing effects. Kastenberger et al. (2012) reported that subcutaneous GPER agonist G1 administration induced anxiety-like behaviors in both intact male and OVX female mice, reducing open-arm exploration in the EPM and time and distance traveled in LDT. Kastenberger and Schwarzer (2014) found that GPER-KO male mice had greater open-arm exploration and increased time in lit areas, indicating reduced anxiety. Further, GPER-KO female mice in estrus displayed more center time, distance traveled, and visits than WT mice in estrus in the OFT. GPER-KO female mice in proestrus also displayed increased center visits, but not center time or distance traveled in the OFT (Kastenberger and Schwarzer, 2014), suggesting an anxiogenic role for GPER.

In contrast, Hart et al. (2014) found that GPER activation with G1 treatment given 30 min prior to testing reduced anxiety behaviors in the EPM in GDE males, suggesting an anxiolytic role for GPER (Hart et al., 2014). This may reflect an impact of hormonal status, or the absence of circulating gonadal hormones in this case, on GPER’s function. Further explorations should investigate whether the presence and levels of circulating gonadal hormones influence GPER’s effects on behavior, possibly through interactions with other estrogen receptors. Hart et al. (2014) also reported that GPER activation differentially enhanced ERK signaling in the DH of female mice, whereas ERα S118 phosphorylation was increased in the ventral hippocampus in male mice (Hart et al., 2014). This highlights sex-dependent responses to GPER activation that may contribute to differences in anxiety behavior.

Stress exposure can alter GPER activity, with increased expression in the amygdala of OVX female mice displaying anxiety-like behavior following acute stress via restraint or forced swimming (Tian et al., 2013). G1 infusions into the basolateral amygdala reversed anxiety-like behaviors, significantly increasing time spent in the EPM’s open arms and time spent in the OF’s center and suggesting anxiolytic GPER effects at this site (Tian et al., 2013). Overall, these findings highlight sex- and site-specific GPER effects and the influence of circulating gonadal hormones. Additional GPER-KO studies are needed to identify specific contingencies leading to GPER’s anxiogenic and anxiolytic properties and to clarify the receptor’s mechanisms in these distinct behavioral effects.

## Animal studies—rats

### GPER’S role in fear behavior

Effects of GPER on fear-related learning and memory in female rats have been understudied; however, evidence in male rats has suggested a critical role for GPER in inhibitory avoidance (IA) memory consolidation. In intact adult male rats, subcutaneous G1 administered immediately, but not 3 or 6 h, after IA conditioning resulted in a longer latency to step down from the platform in a retention test 24 h later (de Souza et al., 2021). GPER also appears to enhance aversive learning and memory consolidation in male rats if activated within a specific time window post-training. Only a higher G1 dose (150 μg/kg) significantly enhanced IA memory, demonstrating the importance of dose in GPER’s effects (de Souza et al., 2021).

### GPER’S role in anxiety behavior

Few studies have investigated sex differences in GPER’s function in anxiety-related behaviors in rats. In GPER-KO female rats, acute restraint stress triggered a greater release of adrenocorticotropic hormone than in WT controls, a response absent in GPER-KO male rats (Zheng et al., 2020). GPER-KO male and female rats also display increased anxiety-like behaviors, demonstrated by a significant reduction in EPM open-arm duration and entries, following SPS. SPS decreased serum corticosterone in WT rats but had no effect in GPER-KO rats (Zheng et al., 2020). These findings suggest that GPER activation may be important for regulating anxiety. Further, intracerebroventricular G1 injections in OVX female rats produced anxiolytic effects, with increased EPM open arm time and decreased closed arm time compared to OVX control rats (Wang et al., 2021). This is likely mediated by rapid PKA signaling, which may distinguish GPER from ERα and ERβ in E2’s influence on anxiety behavior in female rats. Due to the limited number of rat studies, further work is needed to address species differences, discrepancies across animal models, and to determine whether GPER’s anxiolytic effects in rats are sexually dimorphic.

## Human studies

Though very few human studies have investigated GPER and its role in anxiety, existing research suggests its involvement and indicates the translational potential of preclinical findings. Investigations of GPER’s role in patients with generalized anxiety disorder (GAD) have yielded mixed results. While Fındıklı et al. (2016) found serum GPER levels were significantly higher in GAD patients, which correlated with GAD severity, Hurşitoğlu et al. (2025) found significantly decreased GPER levels and no correlation between GPER levels and symptom severity. Fındıklı et al. (2016) excluded women experiencing irregular menstrual cycles, and patients with endocrine disorders and/or receiving drugs influencing serum prolactin levels, whereas Hurşitoğlu et al. (2025) excluded women on hormonal replacement therapy, in post-menopause, and pregnant but did not examine sex differences. Further exploration of GPER influences in GAD are necessary to determine whether these differences in hormonal status might underlie differences in the findings.

Elevated GPER serum levels were also found in patients with major depressive disorder (Findikli et al., 2017) and bipolar disorder (Orhan et al., 2018), but reduced levels in patients with attention deficit hyperactivity disorder (ADHD; Sahin et al., 2018) and autism spectrum disorder (ASD; Altun et al., 2017). Depression and bipolar II are more common in females (Eaton et al., 2012; Diflorio and Jones, 2010), and ADHD and ASD are more prevalent in males (Willcutt, 2012; Napolitano et al., 2022). Thus, variations in GPER levels across psychiatric disorders appear to follow sex-specific patterns.

## Discussion

Despite limited research on GPER’s role in aversive learning and memory, current findings suggest that GPER enhances memory consolidation in both male and female rodents (summarized in Supplementary Table 1). Interestingly, its blockade or elimination leads to sex-specific effects depending on the type of learning and memory. Further, GPER’s effects may also be age-dependent, and the timing of GPER manipulation after emotional learning appears critical for memory consolidation.

GPER’s effects on anxiety behavior have yielded mixed results. Rat studies consistently support an anxiolytic role for GPER, whereas mouse studies show conflicting effects influenced by sex, the presence of circulating hormones, and genetic knockout conditions. These discrepancies point to sex-dependent, and likely hormone-modulated, mechanisms of GPER action, with some downstream effects relying on the JNK pathway and others on the PKA pathway. Additionally, GPER localization within the brain supports its site-specific behavioral effects. Together, these findings call for further research to clarify the mechanisms underlying GPER’s sex and site-specific functions, its role in aversive learning and memory, and critical timing for its most pronounced effects. Addressing these gaps could inform therapeutic strategies targeting GPER for disorders involving emotional dysregulation and anxiety.

### Future directions

Future research should further clarify GPER’s role in fear and anxiety-related behaviors by using selective agonists and antagonists under time-controlled or site-specific experimental conditions. Evidence of sex-specific downstream effects of GPER activation and their influence on anxiety-related and fear behaviors highlights the need to conduct more studies in both sexes. Because hormonal status across the estrous cycle may also affect GPER effects, future studies should also investigate how GPER may interact with classical estrogen receptors and influence downstream signaling pathways. Together, these approaches could provide valuable insight into the therapeutic potential of GPER modulation in treating psychiatric disorders.
