# Supplementary material for: Sex differences in G protein-coupled estrogen receptor-mediated mechanisms in preclinical models of anxiety and fear
Source: Front Behav Neurosci. 2025 Nov 11;19:1655725. doi: 10.3389/fnbeh.2025.1655725 (PMC12644063; doi:10.3389/fnbeh.2025.1655725)
Supplement: Supplementary file 1 [file Table_1.docx]

Supplementary Table 1.

| **Subjects** | **Sex** | **GPER Treatment** | **Behavior** | **Brain** | **Reference** |
| --- | --- | --- | --- | --- | --- |
| ***Mice*** | | | | | |
| 7-week-old mice (20-30g) | Female OVX | GPER KO | Mice were exposed to chronic unpredictable stress with two mild stressors a day, including restraint, wet bedding, 24h of fasting, cold swimming, heating, flatly shaking, or a predator's scent. Chronic unpredictable stress in OVX mice resulted in significantly increased immobility time compared to sham mice, decreased rearing frequency in an OFT, less resting time in the open arm, and prolonged time in the closed arm of an EPM. | N/A | Wang et al., 2021 |
| 12-month-old C57BL/6 mice | Female | Subcutaneous administration of G1 at 1μg/kg, G1 at 5μg/kg, or G15 at 10nmol/mice with G1 at 5μ/kg | Fear memory was tested with fear conditioning through 1 or 3 tone-footshock pairings, followed by contextual memory tests on day 3 using similar contexts as training day, and cue memory tests on day 4 with just the tone. G1 at both doses significantly improved contextual and cue memory in a dose-dependent manner, while G15 blocked the effects. | G1 treatment altered DHPG-LTD in MF-CA3 synapses after G1 treatment and the maintenance of LTD was prolonged after G1 treatment. 3 weeks after viral injection, GFP expression was almost totally restricted to the CA3. GPER expression was significantly up-regulated in the rAdGPR30 group compared with the control. Contextual and cue memory were improved in the rAdGPR30 group but not the rAdGFP group, and DHPG-induced LTD in the CA3 was facilitated in the viral GPR30 expression group. G1 promoted BDNF expression in a dose-dependent manner, and this was blocked by G15 co-application. TrkB phosphorylation was also increased following G1 treatment, while G15 co-application blocked the effects. DHPG significantly reduced GluA expression in slices from G1-treated mice. G1-mediated facilitation of DHPG-LTD was blocked by TrkB and Akt inhibitors. Rapamycin blocked DHPG-induced LTD (MF-CA3) in slices from G1-treated mice. | Xu et al., 2018 |
| 12-month-old and 2-month-old C57BL/6 mice | Male | Subcutaneous administration of G1 at 1μg/kg, G1 at 5μg/kg, or G15 at 10nmol/mice with G1 at 5μ/kg | GPER expression was significantly reduced in the hippocampus of middle-aged mice compared to young adult mice. Spatial memory was tested in a MWM test, fear memory was tested with fear conditioning through 1 or 3 tone-footshock pairings followed by contextual memory tests on day 3 using similar contexts as training day, and cue memory tests on day 4 with just the tone. G1 at both doses significantly improved contextual and cue memory in a dose-dependent manner, while the co-application of G15 blocked the effect of G1. Memory retrieval, represented by time spent in the platform quadrant in a MWM test, was significantly prolonged in G1-treated mice, while the co-application of G15 blocked the effect. G1 in 2-month-old mice did not improve contextual and cued memory, but G15 still reduced memory. | (above) | Xu et al., 2018 |
| 8-week-old C57BL/6 mice | Male GDE | Infusion of G1 at 4 or 8ng/hemisphere or G15 at 1.85 or 7.4ng/hemisphere into the DH | Immediately after OR or OP training, mice were given infusions of vehicle, G1, or G15. Mice given either dose of G1 spent significantly more time with novel objects in object recognition than chance and compared to mice given the vehicle. Only mice given the higher dose of G1 explored the moved object significantly more than chance in object placement, with the mice given the lower dose spending significantly less time than chance with the moved object. Mice given the higher dose of G15 spent chance amounts of time with the novel and moved objects, which was significantly less than the vehicle group. The mice given the lower dose of G15 displayed impaired object placement memory, but object recognition memory was spared. | Previous work with OVX female mice showed elevated levels of phospho-JNK(p46), phospho-JNK(p54) 5 min after DH G1 infusion, and inhibitors of JNK or actin polymerization blocked the memory-enhancing effects of G1, but in this study with GDE male mice, G1 did not increase DH phosphorylation of p46 or p54 JNK. G1 had no effects on p42 or p44 ERK phosphorylation or levels of phospho-PI3K or phospho-AKT.G1 increased phospho-CREB levels in a time-dependent manner. | Machado et al. 2024 |
| 3-8-month-old C57BL/6J mice | Male | GPER KO | There were no significant differences in time spent in, distance traveled, or number of visits to the center of an open field between GPER KO mice and WT. GPER KO mice displayed significantly more ambulation of the open arms in an EPM compared to WT, while the number of open arm visits did not reach statistical significance. In a LDT, GPER KO mice spent significantly more time in the lit area, but distance traveled and visits were not significantly different compared to WT. In the tail-suspension test, no difference in immobility time was observed. In the forced swim test, there was a significant difference in early and late immobility for WT but not GPER KO mice. Stress-induced hypothermia resulted in comparable basal temperature and initial increases in WT and GPER KO mice. WT mice returned to basal after 60 minutes, while the body temperatures of GPER KO mice were still significantly increased. Serum corticosterone levels between the two groups were not significantly different. | N/A | Kastenberger & Schwarzer, 2014 |
| 3 to 8-month-old C57BL/6J mice | Female | GPER KO | In the OFT, GPER KO mice in estrus displayed significantly more center time, distance traveled, and visits than WT mice in estrus. GPER KO mice in proestrus also displayed significantly increased center visits. In the EPM, there were no differences in open arm time, distance, or visits between the two groups. In the LDT, there were no differences in time spent in the lit area, distance traveled, or visits to the lit area. In the tail suspension test, GPER KO mice in diestrus had significantly less immobility time than GPER KO mice in estrus and WT mice in diestrus. In the forced swim test, GPER KO mice in estrus had significantly less early immobility time than WT mice in estrus. Stress-induced hypothermia resulted in comparable basal temperature and initial increases in WT and GPER KO mice. While the body temperature of WT mice dropped significantly lower than basal temperature, the body temperature for GPER KO mice did not. GPER KO mice in diestrus had significantly more serum corticosterone than WT in diestrus, and GPER KO mice in proestrus had significantly less serum corticosterone than WT in proestrus. | N/A | Kastenberger & Schwarzer, 2014 |
| 3 to 8-month-old C57BI/6N mice | Female OVX | Subcutaneous injection of 30 μl of G1 2h before testing | When given G1 2h before testing, mice displayed significantly decreased open arm time, distance, and entries in an EPM test when compared to a control and to mice given agonists for ERα and ERβ. There were no differences in the LDT. In the OFT, mice given G1 2h before testing displayed significantly decreased center time, distance, and entries when compared to a control and to mice given agonists for ERα and ERβ. | N/A | Kastenberger et al., 2011 |
| 3 to 8-month-old C57BI/6N mice | Male | Subcutaneous injection of G1 at 1mg/kg 2h before testing | Mice given G1 displayed significantly decreased open arm time, distance, and entries in an EPM test. In a LDT, mice given G1 also displayed significantly decreased lit-area time, distance traveled, and lit-area entries. Mice given β-oestradiol with G1 displayed significantly decreased lit-area time and distance traveled. In an OFT, mice given G1 displayed significantly reduced center time and distance traveled, while mice given G1 with β-oestradiol displayed significantly reduced center time, distance traveled, and center entries. | N/A | Kastenberger et al., 2011 |
| 8 to 12-week-old C57BL/6J mice | Male GDE | Subcutaneous injection of G1 at 0.3 μg/mouse 30 minutes before each test and before sacrifice | G1-treated mice spent more time and traveled more in the open arms of an EPM compared to the control group but there was no difference between the total number of entries into arms. In the OFT, there was no difference in the time spent in the center, distance in the center, or total distance. | ERK1/2 phosphorylation was not increased in the ventral or dorsal hippocampus at 30 minutes after injection with G1 compared to vehicle. ERα S118 phosphorylation increased with G-1 treatment in the ventral hippocampus. | Hart et al., 2014 |
| 8 to 12-week-old C57BL/6J mice | Female OVX | Subcutaneous injection of G1 at 0.3 μg/mouse 30 minutes before each test and before sacrifice | G1-treated mice did not display any differences in any of the parameters for the EPM or the OFT. | ERK phosphorylation increased in the DH with G1 treatment but ERα S118 phosphorylation did not change in the ventral hippocampus. | Hart et al., 2014 |
| 7 to 8-week-old C57BL mice | Female OVX | Infusion of G1 (0.5 mM, 0.5 μL), G15 (5 mM, 0.5 μL), or a mixture of G1 and G15 into the basolateral amygdala delivered at 0.5 μL/min 8h before testing | Mice given G1 and 2 forced swim stresses prior to testing displayed significantly increased time spent in the open arms of an EPM and significantly more time spent in the central area of an open field. The simultaneous infusion of G15 completely inhibited the effects of G1, with insignificant differences in total arm entries in the EPM and total distance traveled in the open field. G15 decreased the time spent in the open arms or in the central area in the EPM and OFT. | GPER was highly expressed in the BLA and CeA of the amygdala. Expression in the BLA increased in OVX-stressed mice. Acute stress resulted in a significant decrease in the expression of GABAA-α2 and GABAA-γ2 subunits. G1 reduced the downregulation of GABAA-α2 expression in mice undergoing 2 forced swim stresses, while G15 completely inhibited its effects. G1 reduced the downregulation of GABAA-γ2 expression in stressed mice. In controls, G1 did not alter the levels of GPR30, GABAA-α2, and GABAA-γ2, while G15 increased he levels of GPR30 and decreased the levels of GABAA-α2 and GABAA-γ2. G1 reversed the up-regulated expression of GluR1 and reversed the down-regulated expression of NR2B in mice undergoing two forced swim stresses, while G15 completely inhibited these effects. In unstressed mice, G1 did not alter the expression levels of GluR1, p-GluR1-Ser845, NR2A, NR2B, and PSD-95, but G15 increased the expression of GluR1, p-GluR1-Ser845, NR2A, and PSD-95 while decreasing the expression of NR2B. G1 induced the frequency and amplitude of sIPSCs in the BLA pyramidal neurons, but G15 blocked these effects. | Tian et al., 2013 |
| B6.129S6-Gper1tm1Cwan/J mice | Male | GPER KO | In an OFT, no significant differences were observed. In the EPM, however, mice spent significantly more time in the open arms than WT and more frequently transitioned from the closed arms to the open arms. Mice also spent more time in the open arms than WT females. In the MWM, no significant differences were observed between GPER KO mice and WT. In MWM probe trials, mice spent significantly less time in the platform's location than WT 1 week after the last MWM learning trial. Mice also spent significantly less time in the target quadrant than WT females and GPER KO females on day 3. | The CA1 network in the hippocampus appeared significantly more excitable than WT. Paired-pulse ratios were smaller in GPER KO than WT, with significance at interstimulus intervals >100ms. The expression of GluA, an AMPA-receptor subunit, was significantly increased compared to WT. The expression of SNAP25 of both GPER KO and WT were significantly reduced relative to GPER KO females and WT females. | Koitmäe et al., 2023 |
| B6.129S6-Gper1tm1Cwan/J mice | Female | GPER KO | In an OFT, mice in the low E2 stage of estrus spent significantly more time in the periphery of the open field relative to mice in the high E2 stage of estrus. In MWM learning trials, mice took significantly longer than WT to reach the platform on the second day of trials. When splitting the group between high and low E2, the mice with high E2 were the ones taking longer to reach the platform, while the mice with low E2 did not take significantly longer than WT at the same stage. In MWM probe trials, mice in the high E2 stage of estrus spent significantly less time swimming in the location of the platform than WT in the same stage on day 3. GPER KO females altogether spent significantly more time in the target quadrant than GPER KO males. 1 week after the last MWM learning trial, mice in the high E2 stage of estrus spent significantly less time swimming in the platform's location than WT in the low E2 stage, and GPER KO females altogether spent less time in the location than male WT. In contextual fear conditioning, mice froze significantly more than WT and GPER KO males on day 2, when the aversive stimulus was removed. Mice in the high E2 stage froze significantly more than both WT and GPER KO mice in the low E2 stage and WT in the high E2 stage, also on day 2. On day 3 of fear conditioning, no significant differences in freezing were observed. In contextual fear conditioning experiments, mice froze significantly less than GPER KO females on the 2nd day. On day 3, no significant differences in freezing behavior were observed. | The CA1 network appeared to be significantly more excitable in GPER KO high E2 mice relative to WT, but appeared to be significantly less excitable in GPER KO low E2 mice relative to WT. Spinophilin expression was significantly increased in GPER KO low E2 mice relative to WT low E2 and WT high E2. GluA1 expression was significantly increased compared to WT males. SNAP25 expression was significantly increased compared to both GPER KO males and WT males. | Koitmäe et al., 2023 |
| 8 to 10-week-old C57BL/6 mice | Female OVX | G1 infusion at 2 or 4 ng/hemisphere into the DH or 8ng intracerebroventricularly, G15 infusion at 1.85, 3.7, or 7.4 ng/hemisphere into the DH, G15 + E2 at 5μg/hemisphere into the DH or 10 μg intracerebroventricularly, G1 + JNK inhibitor at 1.85ng/hemisphere into the DH, or G1 + ERK inhibitor at 0.5ng/hemisphere | Mice given 4ng/hemisphere of G1 displayed significantly enhanced OR and OP memory. 7.4ng/hemisphere of G15 significantly reduced both OP and OR memory. G1 + vehicle significantly increased OR and OP memory relative to vehicle + vehicle and G1 + G15. G1 + vehicle significantly increased OR and OP memory relative to vehicle + vehicle and vehicle + JNK inhibitor. G1 + ERK inhibitor significantly increased OR and OP memory relative to vehicle + vehicle. Both E2 + JNK inhibitor and E2 + G15 significantly increased OR and OP memory relative to vehicle + vehicle. | GPER does not activate ERK or PI3K/Akt signaling pathways but does increase JNK phosphorylation in the DH. JNK inhibition blocked the effects of GPER activation while ERK inhibition did not. GPER and JNK inhibition did not affect E2 effects on cell signaling in the DH. | Kim et al., 2016 |
| 8 to 10-week-old C57BL/6 mice | Female OVX | Infusions into the DH. G1 infusion at 4ng/hemisphere or 8ng intracerebroventricularly, G15 infusion at 1.85ng/hemisphere, G1 + G15, JNK inhibitor infusion at 2.75ng/hemisphere, G1 + JNK inhibitor, E2 infusion at 5μg/hemisphere or 10μg intracerebroventricularly, E2 + G15, latrunculin A (LatA) infusion at 10 or 50ng/hemisphere, or G1 + Latrunculin A | Mice given latrunculin A at 10ng/hemisphere spent significantly more time than chance with the moved object, while mice given LatA at 50ng/hemisphere spent no more time than chance with the moved object. While the mice given LatA at 10ng/hemisphere also spent a significant amount of time with the novel object in OR, the 50ng/hemisphere group did not. The mice given G1+Vehicle spent significantly more time with the moved object in OP and significantly more time with the novel object in OR than vehicle+vehicle and G1+LatA. | G1 treatment significantly increased CA1 mushroom spine density 40 minutes after infusion. G1 infusion following OR and OP significantly increased total spine and mushroom spine density. A significant increase in cofilin phosphorylation followed G1 treatment at 5 and 15 minutes post-infusion, but levels returned to baseline 30 minutes post-infusion. G1-induced cofilin phosphorylation was significantly higher than levels in the G1+G15 group. The infusion of a JNK inhibitor with G1 blocked the previously seen G1-induced cofilin phosphorylation. Spine density alterations in G1 +G15 and G1+JNK inhibitor groups did not differ, and the increase in spine density with G1 treatment was significantly greater than both groups. The infusion of E2 increased cofilin phosphorylation, and the coinfusion of G15 did not block this effect. LatA, used as an actin polymerization inhibitor, blocked the memory-enhancing effects of G1 in both OP and OR. | Kim et al., 2019 |
| ***Rats*** | | | | | |
| Adult Wistar rats (290-410g) | Male | Subcutaneous administration of G1 at 15, 75, or 150µg/kg after learning, G15 at 100µg/kg, or subcutaneous administration of G1 immediately after retention test at 150µg | G1 administered at the highest dose resulted in a significantly higher recognition index than controls on an OR test, with no significant differences in total time exploring objects during training and retention. When administered 3 and 6 hours after OR, there were no significant differences in training recognition indexes and retention test recognition indexes. Administration immediately after inhibitory avoidance training increased step-down latencies in comparison to controls. When administered 3 and 6 hours after aversive memory training, there were no significant effects. G15 administration resulted in a significantly lower recognition index on an OR test, with no significant differences in total time exploring objects during training and retention. Administration immediately after inhibitory avoidance training resulted in no significant differences in latency to step-down in a long-term retention test when compared to controls. | N/A | de Souza et al., 2021 |
| 10-, 16-, and 22-week-old Sprague Dawley rats | Female | GPER KO (139 bp deletion of GPER gene, Gene ID 171104) generated through CRISPR/Cas9 gene-editing approach | Significant decrease in open-arm time and open-arm visits in an EPM compared to WT across all age groups. Center time, distance, and visits in an OFT were decreased across all age groups compared to WT. | Both mice (who were not involved in testing) and rat brains were studied. There was strong GPER immunofluorescence in the prefrontal cortex, hippocampal formation, amygdala, PVN of the hypothalamus, the intermediate lobe of the pituitary, and the adrenal medulla, but moderate immunofluorescence in the anterior lobe of the pituitary and the adrenal cortex. Tomato+ cells used to tag GPER clustered within the PVN, intermediate lobe of the pituitary, and the adrenal medulla, while sporadic cells were found within the anterior lobe of the pituitary and adrenal cortex. GPER transcripts were detected within the PVN and strong RNAscope signal was detected in the intermediate lobe of the pituitary, prefrontal cortex, basolateral amygdala, and dentate gyrus. Tomato+ neurons were also found in the superior cervical sympathetic ganglion. | Zheng et al., 2020 |
| 10-, 16-, and 22-week-old Sprague Dawley rats | Male | GPER KO (139 bp deletion of GPER gene, Gene ID 171104) generated through CRISPR/Cas9 gene-editing approach | Open-arm time and open-arm visits in an EPM were not significantly different between 10- and 16-week-old male rats and WT, but there were significant decreases in open-arm time and open-arm visits when comparing 22-week-old male rats to WT. This pattern continued in the OFT, with only the 22-week-old rats showing significant decreases in center time and distance. |  | Zheng et al., 2020 |
| 10-week-old Sprague Dawley rats | Female | GPER KO (139 bp deletion of GPER gene, Gene ID 171104) generated through CRISPR/Cas9 gene-editing approach | There was a significant increase in drinking latency and fewer visits compared to WT following a punitive air puff delivered after they first drank water following 20 hours of water deprivation. |  | Zheng et al., 2020 |
| 10-week-old Sprague Dawley rats | Female OVX | GPER KO (139 bp deletion of GPER gene, Gene ID 171104) generated through CRISPR/Cas9 gene-editing approach, subcutaneous administration of E2 at 10µg/kg or G1 at 10µg/kg | OVX resulted in less open-arm time and visits in an EPM and less center time, distance, and visits in an OFT. Administration of E2 or G1 led to a significant increase in center time in the OFT and a significant increase in open-arm time and visits in the EPM. |  | Zheng et al., 2020 |
| 8-week-old Sprague Dawley rats | Female | GPER KO (139 bp deletion of GPER gene, Gene ID 171104) generated through CRISPR/Cas9 gene-editing approach | 2 weeks following single-prolonged stress, female rats displayed a significant reduction in open-arm time and visits in an EPM. They also gained significantly less body weight than WT. Their serum corticosterone level was comparable to the serum corticosterone levels of unstressed female GPER KO rats. |  | Zheng et al., 2020 |
| 8-week-old Sprague Dawley rats | Male | GPER KO (139 bp deletion of GPER gene, Gene ID 171104) generated through CRISPR/Cas9 gene-editing approach | 2 weeks following single-prolonged stress, male rats displayed a significant reduction in open-arm time and visits in an EPM. They also gained significantly less body weight than WT. Their serum corticosterone levels were slightly lower, but not significantly lower, than the serum corticosterone levels of unstressed male GPER KO rats. |  | Zheng et al., 2020 |
| Sprague Dawley rats | Female | GPER KO (139 bp deletion of GPER gene, Gene ID 171104) generated through CRISPR/Cas9 gene-editing approach | Following 30 minutes of acute restraint stress, neither serum corticosterone nor adrenaline levels were significantly different between KO and WT rats, but plasma ACTH was significantly higher in KO rats. |  | Zheng et al., 2020 |
| Sprague Dawley rats | Male | GPER KO (139 bp deletion of GPER gene, Gene ID 171104) generated through CRISPR/Cas9 gene-editing approach | Following 30 minutes of acute restraint stress, neither serum corticosterone nor adrenaline levels were significantly different between KO and WT rats. |  | Zheng et al., 2020 |
| Ten-week-old Sprague Dawley rats | Female | GPER KO (139 bp deletion of GPER gene, Gene ID 171104) generated through CRISPR/Cas9 gene-editing approach | In a MWM test, female KO took significantly longer to find the platform compared to WT during 5-day positioning navigation tests. In the spatial exploration test on the 6th day, they spent slightly less, but not significantly less, time in the target quarter than WT. In an IntelliCage test, they showed similar corner visits during the free exploration period as WT rats, but had fewer corner visits during the 4-day nosepoke learning period. On the first day of nosepoke learning, they had fewer numbers of licks and nosepokes, and they had higher error rates than WT rats during the place and replace learning period. |  | Zheng et al., 2020 |
| Sprague Dawley rats | Male | GPER KO (139 bp deletion of GPER gene, Gene ID 171104) generated through CRISPR/Cas9 gene-editing approach | In a MWM test, male KO took significantly longer to find the platform compared to WT during 5-day positioning navigation tests. In the spatial exploration test on the 6th day, they spent slightly less, but not significantly less, time in the target quarter than WT. In an IntelliCage test, they showed similar corner visits during the free exploration period as WT rats, but had fewer corner visits during the 4-day nosepoke learning period. On the first day of nosepoke learning, they had fewer numbers of licks and nosepokes, and they had higher error rates than WT rats during the place and replace learning period. |  | Zheng et al., 2020 |
| 3-month old Sprague Dawley rats (200-220g) | Female OVX | Intracerebroventricular injection of G1 (50 ng/2 μl/day, 100 ng/2 μl/day, 200 ng/2 μl/day), G15 (75 ng/2 μl/day, 150 ng/2 μl/day), TSPO agonist AC-5216 (100 ng/2 μl/day, 1 μg/2 μl/day), PKA inhibitor fragment (6–22) amide PKI (50 ng/2 μl/day, 100 ng/2 μl/day), or vehicle (0.9% NaCl) | GPER inhibition with the highest dose of G15 increased immobility time in a forced swim test, but both doses reduced rearing frequency in an OFT. No differences were found in the behavior of control and G15-treated rats in an EPM. All 3 doses of G1 significantly reduced the immobility time of rats in forced swimming tests. 100ng G1-treated rats showed increased rearing frequency in an OFT. In the EPM, G1-treated rats spent more time in open arms and less time in closed arms compared to control. AC-5216 significantly decreased immobility time and resulted in rats spending more time in the open arms of an EPM. 100ng AC-5216-treated rats showed more rearing frequency and increased central locomotor activity in an OFT compared to controls. 100ng of PKI pre-injected 30min before G1 administration resulted in reduced rearing frequency and distance traveled in the central area of an OFT while increasing immobility time in forced swimming tests. | The decrease in estrogen resulting from OVX significantly reduced GPER protein levels in the hippocampus. G1 administration elevated mitochondrial membrane potential and increased Superoxide dismutase activity. Higher total antioxidant capacity was found in OVX rats following G1 administration. G1 administration also enhanced PKA and TSPO phosphorylation. | Wang et al., 2021 |
| Adult (~3-month-old) Long Evans rats | Male | G15 infusion at 0.04μg/μL into the perirhinal cortex | Rats given G15 after the sample learning stage had a lower discrimination ratio than rats given vehicle in the choice stage in an object-in-place test under optimal learning conditions, with the rats given 5 minutes in the sample stage and either 20 minutes or 24 hours before the choice stage. In both groups that received 20 minutes and groups that received 24 hours, only rats given vehicle demonstrated a significant preference for the novel object above chance. | Though learning significantly increased JNK phosphorylation relative to non-learning rats, G15 blocked this effect. | Mitchnick et al., 2019 |

**Supplementary Table 1.** Studies on the role of GPER in non-aversive and aversive learning and memory in rodents. Male, female, and gonadectomized mice and rats given different GPER treatments, including GPER KO, administration of G1, and administration of G15, the major findings of each study, and the brain regions studied. OR: object recognition, OP: object placement (spatial memory), EPM: elevated plus maze, OFT: open field test, LDT: light-dark test, MWM: Morris water maze, PVN: paraventricular nucleus, GPER KO: GPER knockout, OVX: ovariectomized, GDE: gonadectomized, DH: dorsal hippocampus

References

de Souza LO, Machado GDB, de Freitas BS, Rodrigues SLC, Severo MPA, Molz P, da Silva JAC, Bromberg E, Roesler R, Schröder N. The G protein-coupled estrogen receptor (GPER) regulates recognition and aversively-motivated memory in male rats. Neurobiol Learn Mem. 2021 Oct;184:107499. doi: 10.1016/j.nlm.2021.107499. Epub 2021 Aug 3. PMID: 34352396.

Hart D, Nilges M, Pollard K, Lynn T, Patsos O, Shiel C, Clark SM, Vasudevan N. Activation of the G-protein coupled receptor 30 (GPR30) has different effects on anxiety in male and female mice. Steroids. 2014 Mar;81:49-56. doi: 10.1016/j.steroids.2013.11.004. Epub 2013 Nov 14. PMID: 24240011.

Kastenberger I, Lutsch C, Schwarzer C. Activation of the G-protein-coupled receptor GPR30 induces anxiogenic effects in mice, similar to oestradiol. Psychopharmacology (Berl). 2012 Jun;221(3):527-35. doi: 10.1007/s00213-011-2599-3. Epub 2011 Dec 7. PMID: 22143579; PMCID: PMC3350630.

Kastenberger I, Schwarzer C. GPER1 (GPR30) knockout mice display reduced anxiety and altered stress response in a sex and paradigm dependent manner. Horm Behav. 2014 Sep;66(4):628-36. doi: 10.1016/j.yhbeh.2014.09.001. Epub 2014 Sep 16. PMID: 25236887; PMCID: PMC4213071.

Kim J, Szinte JS, Boulware MI, Frick KM. 17β-Estradiol and Agonism of G-protein-Coupled Estrogen Receptor Enhance Hippocampal Memory via Different Cell-Signaling Mechanisms. J Neurosci. 2016 Mar 16;36(11):3309-21. doi: 10.1523/JNEUROSCI.0257-15.2016. PMID: 26985039; PMCID: PMC4792941.

Kim J, Schalk JC, Koss WA, Gremminger RL, Taxier LR, Gross KS, Frick KM. Dorsal Hippocampal Actin Polymerization Is Necessary for Activation of G-Protein-Coupled Estrogen Receptor (GPER) to Increase CA1 Dendritic Spine Density and Enhance Memory Consolidation. J Neurosci. 2019 Nov 27;39(48):9598-9610. doi: 10.1523/JNEUROSCI.2687-18.2019. Epub 2019 Oct 18. PMID: 31628182; PMCID: PMC6880457.

Koitmäe A, Karsten Y, Li X, Morellini F, Rune GM, Bender RA. GPER1 deficiency causes sex-specific dysregulation of hippocampal plasticity and cognitive function. J Endocrinol.

2023 Aug 2;258(3):e220204. doi: 10.1530/JOE-22-0204. PMID: 37399525.

Machado GDB, Schnitzler AL, Fleischer AW, Beamish SB, Frick KM. G protein-coupled estrogen receptor (GPER) in the dorsal hippocampus regulates memory consolidation in gonadectomized male mice, likely via different signaling mechanisms than in female mice. Horm Behav. 2024 May;161:105516. doi: 10.1016/j.yhbeh.2024.105516. Epub 2024 Mar 1. PMID: 38428223; PMCID: PMC11065565.

Mitchnick KA, Mendell AL, Wideman CE, Jardine KH, Creighton SD, Muller AM, Choleris E, MacLusky NJ, Winters BD. Dissociable involvement of estrogen receptors in perirhinal cortex-mediated object-place memory in male rats. Psychoneuroendocrinology. 2019 Sep;107:98-108. doi: 10.1016/j.psyneuen.2019.05.005. Epub 2019 May 15. PMID: 31125759.

Tian Z, Wang Y, Zhang N, Guo YY, Feng B, Liu SB, Zhao MG. Estrogen receptor GPR30 exerts anxiolytic effects by maintaining the balance between GABAergic and glutamatergic transmission in the basolateral amygdala of ovariectomized mice after stress. Psychoneuroendocrinology. 2013 Oct;38(10):2218-33. doi: 10.1016/j.psyneuen.2013.04.011. Epub 2013 May 11. PMID: 23669322.

Wang J, Li HY, Shen SY, Zhang JR, Liang LF, Huang HJ, Li B, Wu GC, Zhang YQ, Yu J. The antidepressant and anxiolytic effect of GPER on translocator protein (TSPO) via protein kinase a (PKA) signaling in menopausal female rats. J Steroid Biochem Mol Biol. 2021 Mar;207:105807. doi: 10.1016/j.jsbmb.2020.105807. Epub 2020 Dec 17. PMID: 33345973.

Xu W, Cao J, Zhou Y, Wang L, Zhu G. GPR30 activation improves memory and facilitates DHPG-induced LTD in the hippocampal CA3 of middle-aged mice. Neurobiol Learn Mem. 2018 Mar;149:10-19. doi: 10.1016/j.nlm.2018.02.005. Epub 2018 Feb 6. PMID: 29421611.

Zheng Y, Wu M, Gao T, Meng L, Ding X, Meng Y, Jiao Y, Luo P, He Z, Sun T, Zhang G, Shi X, Rong W. GPER-Deficient Rats Exhibit Lower Serum Corticosterone Level and Increased Anxiety-Like Behavior. Neural Plast. 2020 Aug 28;2020:8866187. doi: 10.1155/2020/8866187. PMID: 32908490; PMCID: PMC7474769.
